# Identifying the critical time points for mental health of asylum seekers and refugees in high-income countries

**DOI:** 10.1017/S204579601900057X

**Published:** 2019-10-02

**Authors:** Domenico Giacco

**Affiliations:** Unit for Social and Community Psychiatry, Queen Mary University of London (WHO Collaborating Centre for Mental Health Service Development), Newham Centre for Mental Health, E13 8SP, London, UK

**Keywords:** Epidemiology, mental health, minority issues and cross-cultural psychiatry, risk factors

## Abstract

**Aims:**

High heterogeneity was found in the prevalence rates of mental disorders in adult asylum seekers and refugees in high-income countries. This may be related to different problems. Among them, there is a changing exposure to risk and protective factors for mental health at different phases of these people's life before migration, and during the migratory journey and resettlement. This study aimed at identifying and distinguishing time points in which distinct risk and protective factors for the mental health of asylum seekers and refugees may occur.

**Methods:**

Systematic review and narrative synthesis. A systematic search was carried out for the period January 2017–August 2019, given the existence of systematic reviews of the evidence up to January 2017.

**Results:**

Two hundred and fifty-two studies were identified with our search and 31 studies were included. The critical time points identified are: (a) before the travel; (b) during the travel; (c) at initial settlement in the host country; (d) when attempting to integrate in the host country; (e) when the immigration status is challenged or revoked. Some factors such as sense of belonging in the host country can be risk factors or protective factors depending on the time point.

**Conclusions:**

These five critical time points can guide the development and selection of well-timed preventive and treatment interventions. They could also be used to stratify samples in epidemiological studies and meta-analyses. At present, we know much more on risk factors than on protective factors. Knowing more about protective factors may inform the development of interventions to foster them.

## Introduction

The recent refugee crisis and migratory waves have been the focus of much political attention in high-income countries (Gianfreda, 2018). A major area of debate has been how to address the health needs of the high numbers of new individuals coming into a country in a short timeframe (Priebe *et al*., 2016).

Refugees’ mental health needs have been a particular concern. Specifically, the factors which may lead to refugees’ migration, i.e. exposure to political persecution and war (UNHCR, 2019) can also predispose them to mental disorders (WHO, 2018).

This gave impetus to the research in this field and a number of studies have assessed prevalence rates in different groups of asylum seekers and refugees and for different mental disorders (e.g. Priebe *et al*., 2016; Turrini *et al*., 2017; Giacco *et al*., 2018).

However, when assessing prevalence rates of mental disorders in these groups, a number of problems may arise, which are reflected by a high heterogeneity in prevalence rates across studies (Priebe *et al*., 2016; Giacco and Priebe, 2018; Giacco *et al*., 2018). These problems are usually summarised as follows:

- There are pragmatic challenges in carrying out epidemiological studies in these groups, such as language barriers, lack of validity of research tools across different cultures (Lewis-Fernandez and Aggarwal, 2013; Brisset *et al*., 2014; Giacco *et al*., 2014) and difficulties in accessing some individuals or groups (Enticott *et al*., 2017); these challenges may lead to bias within studies and methodological artefacts;
- There are differences among individuals and groups of asylum seekers and refugees based on different personal backgrounds, as well as differing social situations in home countries and host countries (Priebe *et al*., 2016; Giacco *et al*., 2018).

The challenges in achieving representative samples can be overcome to some extent by technical safeguards such as using well-trained interpreters or same language interviewers, adopting culturally validated instruments and using carefully designed sampling techniques.

The individual or group differences across asylum seekers and refugees are, instead, true, not influenced by methodology, and, as such, generate unavoidable heterogeneity across studies.

However, there is a third type of problems, which have received less attention from the epidemiological research so far. The statuses of asylum seekers and refugees are determined by legal frameworks and procedures and are transitory in nature. They do not identify stable categories, with which rates of mental disorders can be reliably associated.

These changing statuses can be summarised as follows. All refugees are initially classed as asylum seekers (UNHCR, 2019). The process of obtaining asylum and refugee status can last a long time. During this time, they have the right to remain in the host country but are uncertain as to whether their application will be approved. If the asylum application is not approved, they can leave that country (and move to another country), appeal or become irregular migrants. The refugee status can also be revoked (‘cessation of their refugee status’) (Kapferer, 2003) once a host country court rules that the causes of political persecution or lack of safety (e.g. wars, political regimes, etc.) have ceased in the refugees’ home countries.

So far, refugees, asylum seekers and people who do not have a recognised immigration status in a new country have usually been pooled together in research studies and meta-analyses. This may lead us to ignore differences in stressors which may have a considerable impact on a person's mental health.

The usual stratification of risk factors for mental health is based on a simplified framework in which refugees encounter pre-, peri- and post-migration factors (Priebe *et al*., 2016; Giacco *et al*., 2018). This has not influenced sampling for epidemiological studies as these studies are usually carried out in the host countries (i.e. post-migration).

## Aims

This study aimed to carry out a systematic review of the evidence on risk and protective factors for the mental health of asylum seekers, refugees and in general of people who have been forcibly displaced from their countries. We then sought to classify these factors according to different stages in the life before migration, migration journey and resettlement of these people, with a particular attention to the increasing evidence on post-migration factors (Priebe *et al*., 2016; Miller and Rasmussen, 2017).

## Methods

### Design

This study is a systematic review with a narrative synthesis of the findings.

Previous literature reviews appraised the evidence on risk and protective factors for refugees’ and asylum seekers’ mental health until January 2017 (Priebe *et al*., 2016; Giacco *et al*., 2018).

A systematic search of the evidence was carried out from January 2017 to August 2019 in order to provide the most updated findings. It used similar inclusion criteria but a simplified search strategy and did not include grey literature. This is because our previous reviews were also interested in identifying interventions and good practices, which are often found in other types of documents and sources. The current study, instead, was specifically focused on risk and protective factors for mental health, for which we are aware that an adequate amount of evidence is available in the scientific peer-reviewed literature. Focusing on peer-reviewed literature also ensured that sufficient quality standards were adhered to by all included papers.

### Paper identification and selection

The search words used were the following: (‘refugee’ OR ‘asylum seeker’ OR ‘irregular migrant’ OR ‘undocumented migrant’) AND (‘mental health’) AND (‘risk factors’ OR ‘correlates’). EMBASE, PsychINFO and OVID Medline databases were searched.

Papers were included if: (a) at least 50% of the research participants were refugees and/or asylum seekers; (b) they assessed risk or protective factors for mental disorders; (c) they focused on adult populations (18 or older years of age); (d) they reported on studies carried out in high-income countries (according to the World Bank Classification); (e) they reported on primary research or were literature reviews, excluding opinion pieces; (f) they were published in full articles or full reports in scientific journals, excluding grey literature and conference presentations.

Papers were excluded if they focused only on aspects other than risk and protective factors for mental disorders, e.g. interventions, prevalence rates without correlates, individuals’ experience of care or barriers to access care. Qualitative studies which described patients’ or clinicians’ views about which were potential risks or potential factors for mental health were also excluded. They were deemed as ‘non-focusing on risk and protective factors for mental health’ given that they failed to demonstrate the actual association of those factors with mental health, only suggesting that this may be.

### Procedures and analysis

From each paper identified, risk and protective factors were captured and summarised in an excel extraction table.

The risk and protective factors identified from different studies were initially grouped together according to when they occurred, based on the usual framework (pre-, peri- and post-migration).

The analysis then continued with the aim of re-grouping risk and protective factors in more specific and discrete time points which generated the final time points described in the Results section.

## Results

Our search identified 252 papers in the period January 2017–August 2019. After removal of duplicates and screening according to inclusion criteria, 31 papers were included, i.e. 28 papers reporting on primary research and three literature reviews.

**Fig. 1.**
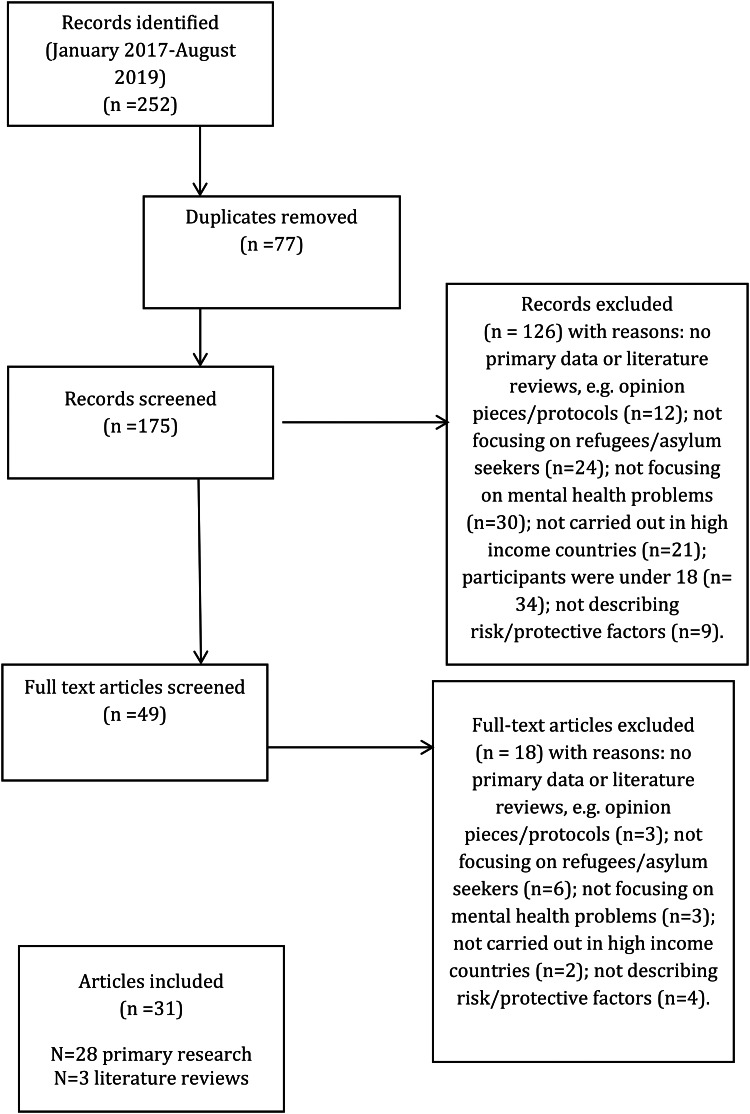
PRISMA flow diagram.

The PRISMA diagram can be found in Fig. 1.

The main characteristics of the included studies are synthesised in Table 1.

**Table 1.** Details of the included studies (*N*  =  31)

Authors	Country(-ies)	Study design	*N* of refugees/asylum seekers included	Risk/protective factors towards mental disorders identified
Anderson *et al*. (2017)	International literature	Systematic review and meta-analysis	N/A	Risk factors: low social support, minority ethnicity, low socioeconomic status, lack of proficiency in host country language and refugee/asylum-seeking status
Beiser and Hou (2017)	Canada	Cross-sectional survey	651 refugees	Risk factors: affiliative feelings towards the home country. Perceived discrimination among men, but not women
				Protective factors: a sense of belonging to host country. Bridging social networks, particularly for women
Ben Fahrat *et al*. (2018)	Greece	Cross-sectional survey	1293 refugees	Risk factor: violent events before, during and post-migration. Uncertainty about asylum application
Chen *et al*. (2017*a*)	Australia	Cross-sectional survey	2399 refugees	Risk factors: both pre-migration potentially traumatic events and post-migration stressors were positively associated with PTSD and severe mental illness. Loneliness and the number of social integration stressors increased the risk of mental health problems particularly if there were pre-migration traumatic events
			762 had PTSD and 394 had other mental illness	
Chen *et al*. (2017*b*)	Australia	Cross-sectional survey	2399 refugees	Protective factors: less financial hardship (economic integration dimension), better English proficiency and self-sufficiency (acculturation dimension), having the capacity to communicate with locals, having friends from different ethnic/religious groups and attending a place of worship weekly or more often (social capital dimension) and feeling welcomed and having a strong sense of belonging in the host country (self-identity dimension)
Dennis *et al*. 2017	Canada	Cross-sectional survey	449	Risk factors: experiencing abuse, pain post-birth, worried about family members left behind, had food insecurity and had reduced access to healthcare
			Refugee women who had recently given birth (2–16 weeks before)	
				Protective factors: higher levels of social support and feeling they belonged to a community
Euteneuer and Schäfer (2018)	Germany	Cross-sectional online survey	164 male refugees	Risk factors: downward mobility in subjectively perceived social status is associated with depression
Finnvold and Ugreninov (2018)	Norway	Analysis of register data	30 871 refugees	Protective factors: ethnic density effect. Refugees living in clusters tend to have less use of mental healthcare services (proxy for mental health problems)
Hynie, 2018	International literature	Narrative literature review	N/A	Risk factors: exposure to violence and trauma, particularly repeated exposures and extreme violence such as torture, an increased length of displacement
Hvidtfeldt *et al*. (2019)	Denmark	Analysis of register data	46 104 refugees	Risk factors: long asylum-decision waiting periods were associated with an increased risk of psychiatric disorders
Kaltenbach *et al*. (2018)	Germany	Cohort study	57 refugees – 1 year follow-up	Risk factors: a higher number of traumatic experiences was related to a greater intensity of PTSD symptoms. In addition, post-migrational stressors were associated with a worsening of PTSD symptoms over the course of the year. Emotional distress was associated with current negative life events, unemployment and frequent visits to physicians
Kartal *et al*. (2018)	Australia and Austria	Cross-sectional survey	138 refugees	Risk factors: exposure to trauma and acculturative stress mediating the effect of traumatic exposure
Kashyap *et al*. (2019)	USA	Cohort study	323 refugees who are torture survivors	Protective factors (after 6 months of treatment): female gender, change to a more secure visa status, accessing more social services, not reporting chronic pain, stable housing and employment
Le *et al*. (2018)	Switzerland	Cross-sectional survey	108 refugees and asylum seekers having experienced or witnessed torture	Risk factors: perceived uncontrollability and distress during torture might be significant risk factors for current mental health of torture survivors
Lee *et al*. (2017)	South Korea	Literature review	N/A	Risk factors: traumatic experiences, longer stay periods in third country, forced repatriation, acculturative stress, low income, older age, poor physical health
				Protective factors: educational level, social support, family relationship quality and resilience
Leiler *et al*. (2019)	Sweden	Cross-sectional survey	510 refugees, among them 367 asylum seekers	Risk factors: waiting for decision on asylum
Miller *et al*. (2018)	USA	Cross-sectional survey	165 refugees	Risk factors: separation from family members
Müller *et al*. (2018)	Germany	Cross-sectional survey	620 migrants, 79 asylum seekers	Risk factors: asylum seeking (as opposed to having achieved permanent residence status)
Mulugeta *et al*. (2019)	USA	Analysis of register data	1055 refugees	Risk factors: female gender, longer displacement
Myhrvold and Småstuen (2017)	Norway	Cross-sectional survey	90 undocumented migrants, 48 migrating because of war or persecution	Risk factors: having experienced abuse. Having family and work had no positive effect on mental health
Nickerson *et al*., (2017)	Switzerland	Cross-sectional survey	134 refugees	Risk factors: female gender, age, time in Switzerland, trauma exposure and post-migration stress
Nosè *et al*. (2018)	Italy	Cross-sectional survey	109 male asylum seekers or refugees	Risk factors: time after departure, length of stay in the host country and number of traumatic events
Park *et al*. (2018)	South Korea	Cross-sectional survey	109 refugees	Risk factors: early traumatic experiences were positively associated with depressive symptoms
Poole *et al*. (2018)	Greece	Cross-sectional survey	135 asylum seekers and refugees in a refugee camp	Risk factors: female sex, number of children and increased time in the asylum process in Greece were significant risk factors for major depressive disorder
				Protective factors: being married
Rees *et al*. (2019)	Australia	Cross-sectional survey	1335 women accessing public antenatal clinics (685 from conflict-affected backgrounds)	Refugees were more likely than host country natives to present with major depressive disorder and to have been exposed to potentially traumatic events, intimate partner violence. They had less social support and more financial stressors
Sangalang *et al*. (2018)	USA	Cross-sectional survey	3257 immigrants, of whom 1620 are refugees	Risk factors: pre-migration potentially traumatic events, discrimination, acculturative stress and family conflict
Schick *et al*. (2018)	Switzerland	Cohort study	71 asylum seekers/refugees who have been in psychiatric treatment – 3 year follow-up	Improvement in post-migration difficulties, particularly visa status, was associated with PTSD, depression and anxiety symptom improvement
Schweitzer *et al*. (2018)		Cross-sectional survey	104 female refugees	Risk factors: higher numbers of trauma events and post-migration living difficulties, having children, age
Song *et al*. (2018)	USA	Cross-sectional survey	278 refugees and asylum seekers exposed to torture	Risk factors: female sex, older age, cumulative exposure to multiple torture types, unstable housing and time spent in the USA before presenting for services
Tinghög *et al*. (2017)	Sweden	Cross-sectional survey	1215 refugees	Risk factors: potentially traumatic events, especially being exposed to interpersonal violence, and post-migration stress (composite measure including perceived discrimination, lack of host country-specific competencies, economic strain, loss of home country, home country and family concerns, social strains and family conflicts)
Yang and Mutchler (2019)	USA	Cross-sectional survey	127 refugees older than 55 years.	Risk factors: self-reported poor health
				Protective factors: larger household size and older age of arrival into the USA

Five time points were identified, in which all the risk and protective factors identified can be categorised. These time points are: (a) before travel; (b) during travel; (c) initial settlement in the host country; (d) integration in the host country; (e) challenges to or revocation of the immigration status.

**Fig. 2.**
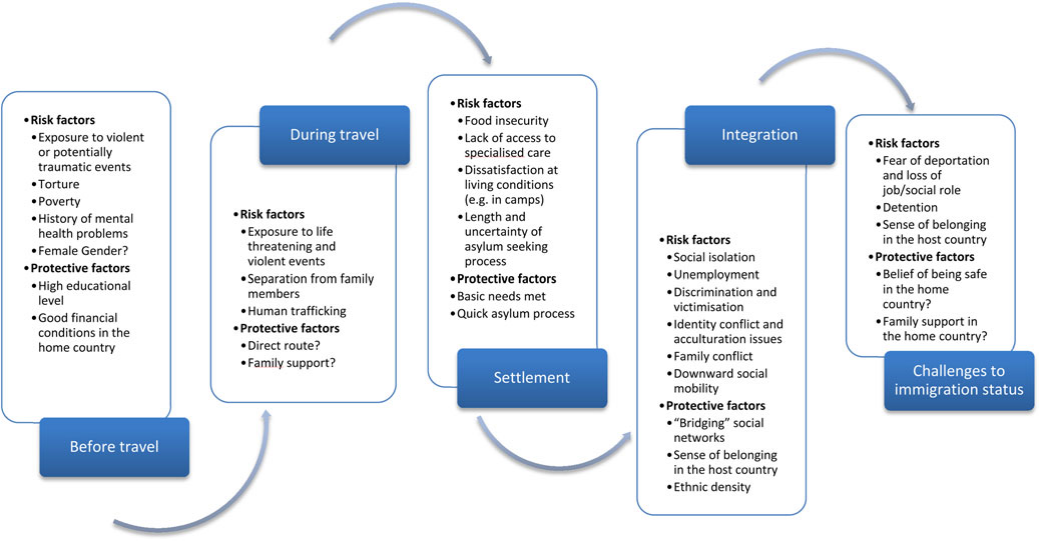
Critical time points for the mental health of asylum seekers and refugees and risk and protective factors at play.

A summary of risk and protective factors at all of these time points can be found in Fig. 2.

These different time points can all be experienced by individuals who are forced to migrate from their countries. However, this is not necessarily the case for all of these individuals. For example, some of them may have their asylum applications rejected and never reach the status of a refugee. Others may integrate successfully and, hence, remain in the host country as naturalised citizens without their residence status being challenged.

### Before travel

Exposure to potentially traumatic events (Chen *et al*., 2017*a*; Myhrvold and Småstuen, 2017; Giacco *et al*., 2018; Hynie, 2018; Kartal *et al*., 2018; Park *et al*., 2018), violent events witnessed or experienced (Ben Fahrat *et al*., 2018; Kaltenbach *et al*., 2018, Schweitzer *et al*., 2018; Rees *et al*., 2019) and particularly being subjected to torture (Nickerson *et al*., 2017; Le *et al*., 2018; Song *et al*., 2018) have all been linked to high likelihood of developing post-traumatic stress disorder and other forms of psychological distress. This is more likely if the experience of torture is accompanied by intense emotions of anger, fear and perceived uncontrollability (Le *et al*., 2018). The risk of developing a mental disorder also increases if a person is exposed to multiple potentially traumatic events and/or if post-migration adversities occur in addition to traumatic events before migration (Chen *et al*., 2017*a*; Lee *et al*., 2017; Nickerson *et al*., 2017; Tinghög *et al*., 2017; Giacco *et al*., 2018; Kartal *et al*., 2018; Nosè *et al*., 2018; Sangalang *et al*., 2018; Schweitzer *et al*., 2018; Song *et al*., 2018).

As well as traumatic events, exposure to economic hardship and social deprivation in home countries and a history of mental health problems before migration can predict mental disorders after settlement in host countries (Anderson *et al*., 2017; Giacco and Priebe, 2018).

On the other hand, having experienced good financial conditions and having attained a higher level of education in the home country are associated with reduced rates of mental disorders after migration (Lee *et al*., 2017; WHO, 2018).

Some studies have found that female gender is associated with a higher likelihood of presenting clinically significant mental health symptoms in asylum seekers and refugees (Poole *et al*., 2018; Song *et al*., 2018; Mulugeta *et al*., 2019). However, most studies did not identify differences across genders (see Table 1).

### During travel

Refugees travel through dangerous land routes or via sea and can be exposed to violent events or abuse during their journey. Exposure to violent events during migration (Ben Fahrat *et al*., 2018; Poole *et al*., 2018) and to human trafficking (Ottosova *et al*., 2016; WHO, 2018) have been linked with poor mental health after resettlement. Separation from family members as part of the migration or during the migration (Miller *et al*., 2018) also has been found to have negative consequences on mental health.

There are no studies which have explored protective factors during migration. However, it is likely that travelling through less dangerous or more direct routes and having the support of family members or trusted friends during the travel is associated with less stress and impact on mental health.

### Settlement in a host country

The risk factors encountered in the first period after settlement in a host country are related to food insecurity, lack of accommodation (Dennis *et al*., 2017; Song *et al*., 2018) and dissatisfaction with accommodation, particularly in refugee camps (Poole *et al*., 2018). A longer duration of the asylum seeking process and lack of information can generate prolonged stress and uncertainty which was found to be associated with clinically relevant psychological symptoms (Ben Fahrat *et al*., 2018; Poole *et al*., 2018).

Better conditions of settlement, access to health care (Dennis *et al*., 2017) and a prompter and/or smoother process for obtaining a residence status can have a positive impact on the mental health of refugees (Müller *et al*., 2018; Kashyap *et al*., 2019). The uncertainty related to asylum decisions is a factor which has been found to be consistently associated with poor mental health in a number of studies (Ben Fahrat *et al*., 2018; Leiler *et al*., 2019; Hvidtfeldt *et al*., 2019).

### Integration in a host country

Prevalence studies show higher rates of mental disorders (particularly anxiety and depressive disorders) in long-term resettled refugees (at least 5 years) than in those at first resettlement (Bogic *et al*., 2015). Recent studies have also shown that an increased length of displacement can be associated with an increased likelihood of experiencing mental disorders (Hynie, 2018; Nosé *et al*., 2018; Mulugeta *et al*., 2019).

During the process of integration in the host country, risk factors for mental health can be social isolation (Anderson *et al*., 2017; Beiser and Hou, 2017; Chen *et al*., 2017*a*; Lee *et al*., 2017; Hynie, 2018; Schweitzer *et al*., 2018), unemployment (Kaltenbach *et al*., 2018), discrimination and victimisation (Sangalang *et al*., 2018), struggles related to their cultural identity and acculturation issues (Chen *et al*., 2017*b*; Lee *et al*., 2017) and downward social mobility (Euteneuer and Schafer, 2018).

A protective factor for mental health is having ‘bridging social networks’ (Chen *et al*., 2017*b*; Beiser and Hou, 2017), i.e. social networks including people from different ethnic groups. Developing a sense of belonging in the host country was also found to be associated with reduced likelihood of mental disorders (Chen *et al*., 2017*b*; Myhrvold and Småusten, 2017; Beiser and Hou, 2017) and so were positive changes in post-migration living arrangements (Shick *et al*., 2018). In particular, achieving a visa or resident status was associated with better response to treatment (Schick *et al*., 2018). Findings also suggest that refugees living in areas where there is a high number of people from their own ethnic group, i.e. a high ‘ethnic density’ may be less likely to use mental health services and hence less likely to present severe mental health needs (Finnvold and Ugreninov, 2018). In accordance with this, a larger household size was found to be a protective factor for mental health (Yang and Mutchler, 2019).

### Immigration status challenged or revoked

At this time point, threats of deportation, detention in immigration centres and losing jobs or accommodation are recognised risk factors for psychological distress and diagnosable mental disorders (Priebe *et al*., 2016; Kashyap *et al*., 2019). A sense of belonging in the host country, which is a protective factor during integration, can become a risk factor if the immigration status is challenged (Lee *et al*., 2017).

No studies have assessed protective factors when immigration status is challenged or revoked. It might be expected that a sense of being safe in the home country, having a family or a social network there and maintaining a strong sense of belonging to the home country may be protective against psychological distress (whilst ‘affiliative feelings towards the home country’ can be a risk factor for mental health during the process of integration in the host country, as found in Beiser and Hou, 2017).

## Discussion

### Main findings

This review has identified five time points at different stages of the migration process of asylum seekers and refugees in which different risk and protective factors may occur. The time points identified go beyond the usual and simplified distinction of pre-migration, peri-migration and post-migration risk factors and constitute a more comprehensive categorisation.

### Implications

The risk and protective factors identified have all been variably described in previous studies (Lindencrona *et al*., 2008; Steel *et al*., 2009; Bogic *et al*., 2012; Bogic *et al*., 2015). This study adds a novel categorisation with five distinct time points in which risk and protective factors can be encountered by refugees after migration. Despite the increased complexity of this categorisation, it may have a number of advantages as a framework for research, psychosocial assessment and selection of interventions.

First, this more comprehensive categorisation may help in interpreting the heterogeneity of prevalence studies (Bogic *et al*., 2015; WHO, 2018). It can provide a useful way of stratifying research participants in more homogeneous groups with regards to risk and protective factors for their mental health, and these groups may show more similar prevalence rates of mental disorders.

Second, the risk and protective factors identified can be a target of interventions aiming to address or foster them in prevention and treatment programmes specifically provided at different time points.

Third, it is important to observe that some factors may act as either risk or protective factors depending on the different time points. For example, sense of belonging in the host country is a protective factor during integration (Chen *et al*., 2017*b*; Myhrvold and Småusten, 2017; Beiser and Hou, 2017), but a risk factor in case of immigration status being challenged or revoked (Geraci (2011) and Lee *et al*., 2017). Similarly, affiliative feelings towards the home country can be a source of anxiety during migration, settlement or integration in the host country (Beiser and Hou, 2017), but are likely to represent a protective factor in case of the possibility of repatriation. Interventions and psychosocial assessments should take the variation in the role of these factors into account.

Finally, decision making about the type of interventions to prevent or alleviate psychological distress can be informed by the critical time points identified. Addressing basic needs and accommodation problems may be more important and effective during the first resettlement than embarking in complex psychological or pharmacological interventions. If the immigration status is challenged or revoked, supportive interventions to understand and address concerns and fears about repatriation or to help people plan their return may be provided, particularly if deportation is inevitable (Geraci, 2011). Standard psychiatric interventions may not address these specific concerns and be ineffective, if such fears are a core reason for anxiety or disturbances in mood.

### Limitations

The following limitations need to be acknowledged:

- our findings are based on an appraisal of research studies. These studies may not have been able to reach people who face barriers to access mental health services or research teams (Enticott *et al*., 2017);
- the effect of risk and protective factors on rates of mental disorders were not analysed quantitatively using a meta-analysis. Hence we are unable to speculate on which of them has the highest effect on mental health and should be prioritised. However, a meta-analysis of individual risk factors was carried out previously, reporting high heterogeneity and variable methodological quality of the included studies (Steel *et al*., 2009). In this study, the aim was to identify when risk and protective factors are likely to occur and this can also be of importance for planning treatments and prevention strategies.
- a general consideration of risk factors, pooling all mental disorders together, is likely to emphasise risk factors for the most frequently diagnosed disorders (e.g. anxiety and depressive disorders) with less of a focus on the factors associated to disorders with comparatively lower prevalence rates (e.g. psychotic disorders, somatisation disorders, etc.) (Giacco *et al*., 2018).
- low- and middle-income countries were excluded because it was felt that examining the risk and protective factors for mental health in these countries would justify a specific review study. There is an increasing amount of studies exploring the mental health of asylum seekers and refugees in low- and middle-income countries. Twenty-three papers have been written in this area in the last two and a half years and had to be excluded from this review (see Fig. 2). Moreover, in low- and middle-income countries, additional factors might intervene which may complicate the categorisation proposed. A particular issue is that some of these countries can be deemed as ‘transit countries’. Migrants expect to only spend some time in transit countries in preparation of reaching the planned final destinations. Recent studies have found high rates of mental disorders in transit countries (Arsenijevic *et al*., 2017; Vukčević Marković *et al*., 2017; WHO, 2018) and specific studies are required to understand in detail why this is. Moreover, a difficult financial situation in the host country might make the process of integration of refugees even more difficult and challenging.

### Suggestions for future research

There are a number of unresolved questions in this area, which may be addressed by future research.

All refugees, by definition, have been exposed to stressful events such as political persecution, war and/or torture (UNHCR, 2019). Yet, many of them will not present with diagnosable mental disorders. Future studies should focus on understanding how these individuals are able to show such resilience and use the mechanisms and factors underlying resilience to inform interventions.

With regard to high-income countries, there is a need to better understand the potential selection processes which may occur during migration (WHO, 2018). There may be differences in socio-demographic characteristics among refugees who migrate to high-income countries and those who stay in the home country or migrate to other poorer or nearer countries. Selection processes may also occur between people who stay for a longer time in a given host country and those who leave that country after a shorter period of time.

Finally, understanding the impact of changing social contexts and political climates on the equilibrium between post-migration risk and protective factors may need to be in the research agenda too. Increasing negative (or, on the other hand, positive) social attitudes in host countries may influence the level of discrimination that refugees encounter, the investment in social integration programmes and the opportunities for refugees to flourish. This, in turn, could have a negative impact on asylum seekers’ and refugees’ mental health. Interventions provided within mental health services may not be able to address these issues on their own.
